# Synthesis of Cu_2_O-Modified Reduced Graphene Oxide for NO_2_ Sensors

**DOI:** 10.3390/s21061958

**Published:** 2021-03-11

**Authors:** Manman Huang, Yanyan Wang, Shuyang Ying, Zhekun Wu, Weixiao Liu, Da Chen, Changsi Peng

**Affiliations:** 1 School of Optoelectronic Science and Engineering & Collaborative Innovation Center of Suzhou Nano Science and Technology, Soochow University, Suzhou 215006, China; 20195239010@stu.suda.edu.cn (M.H.); 20195239037@stu.suda.edu.cn (S.Y.); 20185208036@stu.suda.edu.cn (Z.W.); 20174208118@stu.suda.edu.cn (W.L.); changsipeng@suda.edu.cn (C.P.); 2 Key Lab of Advanced Optical Manufacturing Technologies of Jiangsu Province & Key Lab of Modern Optical Technologies of Education Ministry of China, Soochow University, Suzhou 215006, China; 3 College of Electronics, Communications, and Physics, Shandong University of Science and Technology, Qingdao 266590, China; chenda@sdust.edu.cn

**Keywords:** reduced graphene oxide (rGO), cuprous oxide nanowires, polypyrrole, gas sensors, NO_2_ sensing, p-type semiconductor

## Abstract

Nowadays, metal oxide semiconductors (MOS)-reduced graphene oxide (rGO) nanocomposites have attracted significant research attention for gas sensing applications. Herein, a novel composite material is synthesized by combining two p-type semiconductors, i.e., Cu_2_O and rGO, and a p-p-type gas sensor is assembled for NO_2_ detection. Briefly, polypyrrole-coated cuprous oxide nanowires (PPy/Cu_2_O) are prepared via hydrothermal method and combined with graphene oxide (GO). Then, the nanocomposite (rGO/PPy/Cu_2_O) is obtained by using high-temperature thermal reduction under Ar atmosphere. The results reveal that the as-prepared rGO/PPy/Cu_2_O nanocomposite exhibits a maximum NO_2_ response of 42.5% and is capable of detecting NO_2_ at a low concentration of 200 ppb. Overall, the as-prepared rGO/PPy/Cu_2_O nanocomposite demonstrates excellent sensitivity, reversibility, repeatability, and selectivity for NO_2_ sensing applications.

## 1. Introduction

NO_2_, as a major air pollutant, is responsible for acid rain and hazardous to human respiratory tracts. According to the World Health Organization (WHO), the safety limit for NO_2_ gas is 410 ppb per hour [1]. Hence, monitoring the trace amounts of NO_2_ is necessary from health perspective and plays an important role in environmental pollution [2], air quality, and industrial safety [3,4,5,6,7].

The two-dimensional graphene, discovered by Novoselov et al. in 2004 [8], is widely employed as a promising sensing material due to its high specific surface area (2.6 × 10^3^ m^2^/g) [9,10,11,12,13], ultra-high room-temperature electron mobility (2.0 × 10^5^ cm^2^/Vs), and chemical stability [14,15]. Additionally, graphene can be easily and cost-effectively prepared by a wide range of techniques, such as mechanical peeling [8], chemical vapor deposition [16,17], silicon carbide (SiC) epitaxial growth [18], redox method [19], and other methods. It has been reported that the changes in the external chemical environment result in significant differences in the sensing performance of graphene [20]. Schedin et al. have first reported the performance of graphene-based gas sensors in 2007 [21], however, the as-prepared sensors exhibited distinct disadvantages, such as slow as well as low response and poor selectivity [22,23].

Similar to graphene, metal oxides (MO) can also be used as sensing materials; however, MO-based sensors also possess some defects. The first MO-based commercial sensor appeared in the 1960s [24]. Moreover, the operating temperature of MO-based sensors ranges from 150 to 400 °C and such a high operating temperature raises safety concerns, degrades device stability and reduces the operating life [22,25,26,27,28,29,30]. Some efforts have been made to achieve room-temperature sensing [24,31]. Currently, graphene-based nanocomposites are the focus of research for sensing applications [32,33,34,35]. In particular, MO-graphene nanocomposites have garnered intensive attention because of their excellent sensing properties [14]. Cu_2_O, as a typical p-type semiconductor, is a promising candidate among different metal oxides. Different morphologies of Cu_2_O, such as spherical, rod-like, lamellar, and tubular, have been studied for sensing applications [36,37,38,39,40]. It is expected that the incorporation of Cu_2_O between graphene nanosheets can enlarge the specific surface area, increase active sites and enhance the adsorption capacity of graphene, improving the affinity for gas molecules. Additionally, the presence of Cu_2_O can prevent the restacking of graphene sheets and overcome inferior gas selectivity of graphene [41,42].

Compared with common polymers, conductive polymers possess a unique unlocalized conjugated π-electron system [43]. The long-range conjugation not only greatly reduces the gap between the bonding and antibonding bands, but also widens the distance between two bands. It increases the number of orbitals in the band and reduces the gap between orbitals, allowing the free movement of carriers within the band. Polypyrrole (PPy), as an important conductive polymer [44], renders high chemical stability, high conductivity, redox reversibility, good dispersion, simple preparation, and low cost [45,46], showing great potential in sensing applications [46]. By introducing PPy into graphene-based materials, the electrostatic repulsion between PPy nanoparticles can effectively prevent the accumulation of graphene sheets, optimizing the sensing properties of graphene-based materials.

The comparison of NO_2_ sensors, based on reduced graphene oxide (rGO) or Cu_2_O composites, reveals that designing and fabricating sensing devices based on binary or ternary components with excellent sensing properties is still a challenge (Table 1) [12,14]. Hence, in this work, graphene-polypyrrole-coated copper oxide nanowires ternary components were designed and prepared for room temperature for sensing applications. The PPy/Cu_2_O were easily formed by the hydrothermal method using pyrrole as templates. Further assembly of graphene oxide (GO) with PPy/Cu_2_O and reduction were carried out to form ternary components by optimizing the preparation conditions, where the micro- and nano-scale of each component was regulated and combined with the optimal composite ratio to obtain the composite nanomaterials with specific properties. Moreover, the synergistic reinforcement between different components leads to optimal performance. In general, the as-prepared gas sensors via assembly techniques realize room-temperature sensing. These sensors exhibit a maximum NO_2_ response of 42.5% and are capable of detecting NO_2_ at a low concentration of 200 ppb. In addition, the sensors show excellent repeatability and selectivity.

## 2. Experimental

### 2.1. Materials

All chemical reagents were of analytical grade and used as received without further purification. Pyrrole, acetic acid, ethanol, acetone, concentrated sulphuric acid, and hydrogen peroxide were purchased from the Sinopharm Chemical Reagent Co., Ltd., Shanghai, China. Copper acetate monohydrate was obtained from the Gretel Pharmaceutical Technology Co., Ltd., Suzhou, China.

### 2.2. Synthesis of Polypyrrole-Coated Cu_2_O Nanowires

PPy-coated Cu_2_O nanowires were prepared by a one-step hydrothermal method [47,48], where copper acetate monohydrate was used as a precursor and pyrrole acted as a reducing and structure-directing agent under weak acidic conditions. Initially, 0.2 g of copper acetate monohydrate were added in a certain amount of deionized water and ultrasonicated for 5–10 min for complete dissolution. Then, the pyrrole monomer (0.075 mL) was added into a small amount of deionized water. After transient ultrasonication, it was slowly added to the above solution and stirred with a glass rod. Then, 0.15 mL of acetic acid (1 mol/L) was added to the above mixture to make sure the dissolution of pyrrole monomers. The resulting mixture was transferred to an autoclave and placed in an oven at 120 °C for 12 h. Finally, the reaction solution was cooled down to room temperature and polypyrrole-coated copper oxide nanowires were obtained by sequentially washing with deionized water, ethanol, and acetone, followed by filtration and drying. The hydrothermal temperature of 120, 140, and 160 °C was also used to investigate the influence of growth temperature on structure and morphology.

### 2.3. Preparation of rGO/PPy/Cu_2_O Nanocomposites

GO was prepared by the modified Hummers method [49]. First, a certain amount of PPy/Cu_2_O nanowires was added into ethanol and ultrasonicated for 30 min to obtain a concentration of 2 mg/mL. A certain amount of 2 mg/mL GO solution was added into the above solution, ultrasonicated for 30 min and magnetically stirred for 3–5 h. Finally, the completely dispersed and uniformly mixed solution was filtered, washed, and dried to obtain GO/PPy/Cu_2_O nanocomposite. The as-prepared GO/PPy/Cu_2_O nanocomposites were placed in a tube furnace and heated at 350 °C for 1 h under the protection of Ar gas, resulting in the reduction in GO and formation of rGO/PPy/Cu_2_O nanocomposites.

### 2.4. Fabrication of rGO/PPy/Cu_2_O-Based Gas Sensor

**Figure 1:**
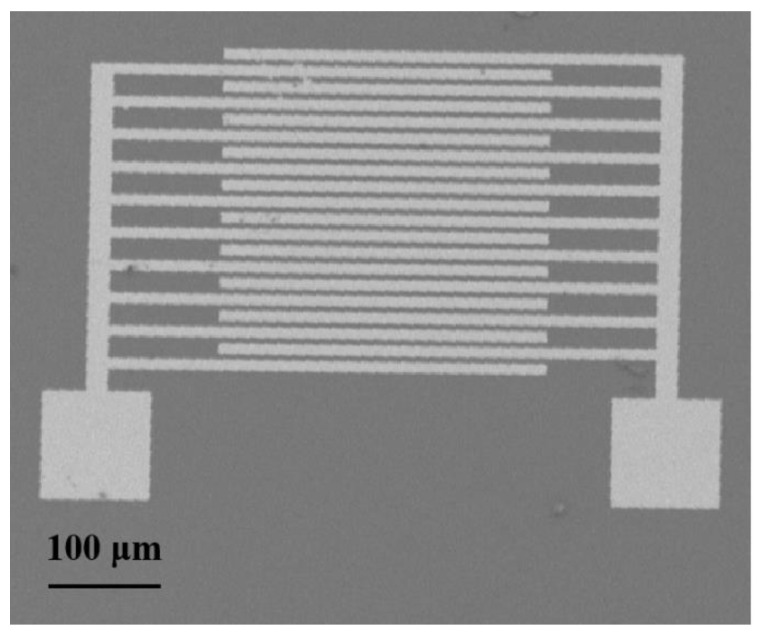
SEM image of the tested device.

Herein, the interdigitated electrode for gas sensing was prepared by the lift-off process [13]. First, a silicon wafer was heated at 90 °C in a mixture of concentrated H_2_SO_4_ and H_2_O_2_ for a certain time to obtain a hydrophilic silicon substrate. Then, the photoresist was applied to the silicon substrate. After a series of operations, we have obtained the planar interdigital electrodes by using the self-designed mask plate after exposure, sputtering and peeling. The as-prepared rGO/PPy/Cu_2_O nanocomposite was ultrasonically dispersed in ethanol to obtain a suspension with a concentration of 1 mg/mL. The same amount of dispersion was measured by a micropipette and applied to the interdigital electrode. The two poles of the interdigital electrode were connected through the gas sensing material. To observe it visually, SEM characterization of the device is shown in Figure 1. Finally, the as-prepared sensor was vacuum-dried before further characterization.

### 2.5. Gas Sensor Sensitivity

**Figure 2:**
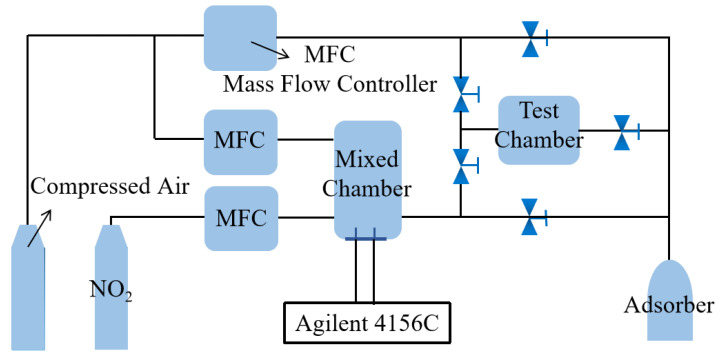
Schematic illustration of the gas sensing system.

In order to simulate the real detection environment, we have utilized compressed air as a background and dilution gas during the gas sensitivity test. The test temperature was set at 25 °C and mild test conditions were used, which are comparable to the practical applications. Figure 2 shows a sketch of the gas sensing set-up. Prior to the test, the background gas was introduced for a certain time to remove the residual exhaust gas from the gas path. Then, the desired concentration of NO_2_ gas was introduced at the beginning of the test. The NO_2_ concentration (C_N_) can be given as:

(1) $$C_{N}\;=\frac{5000\;\times {\;F}_{N}}{F_{N}+{\;F}_{C}}$$

The concentration of the NO_2_ cylinder was 5000 ppm. F_N_ (sccm) refers to the flow rate of the NO_2_ gas and F_C_ (slm) represents the flow rate of diluted gas (air).

When NO_2_ gas passed through the sensor, the hole concentration of as-prepared rGO/PPy/Cu_2_O nanocomposite increased and the resistance decreased due to the adsorption between NO_2_ gas and as-prepared rGO/PPy/Cu_2_O nanocomposite. The gas sensitivity (S, %) can be calculated from the change in resistance using the I-T curve, as given below:

(2) $$S(\%)\;=\;\frac{R_{g}\;-{\;R}_{a}}{R_{a}}\;\times \;100\%\;=\;\frac{\Delta R}{R_{a}}\;\times \;100\%$$

where R_a_ represents the resistance of sensor in air and R_g_ corresponds to the resistance after the introduction of the NO_2_ gas.

## 3. Results and Discussion

### 3.1. Characterization of as-Prepared Nanocomposites

**Figure 3:**
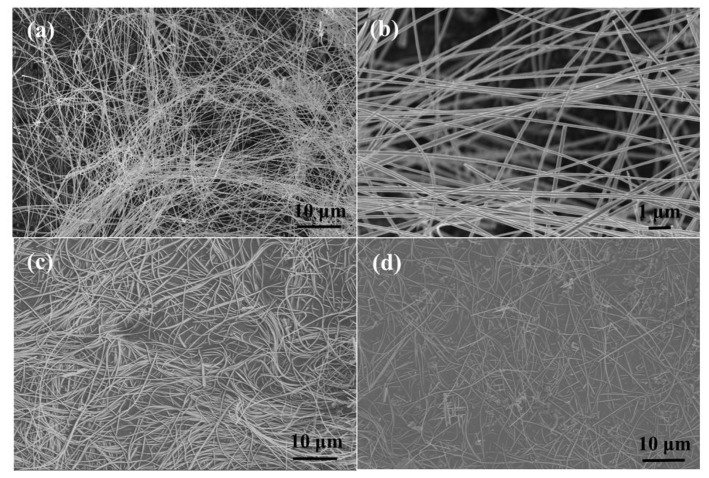
SEM images of hydrothermally-prepared PPy/Cu_2_O nanowires at different temperatures: (**a**,**b**) 120 °C; (**c**) 140 °C; and (**d**) 160 °C.

Scanning electron microscopy (SEM) is employed to explore the influence of different growth temperatures on the morphology and microstructure of PPy/Cu_2_O nanowires. As shown in Figure 3a, the length of copper oxide nanowires, grown at 120 °C, ranges from tens to hundreds of microns. Additionally, a smooth surface with uniform thickness is achieved (Figure 3b). However, when the growth temperature is increased to 140 °C, the copper oxide nanowires started to bend and exhibited different lengths (Figure 3c). One should note that the shorter copper oxide nanowires are not desirable for subsequent preparation of conductive films. As shown in Figure 3d, the further increase in growth temperature to 160 °C resulted in shorter copper oxide nanowires. Hence, the lower growth temperature is more favorable for copper oxide nanowires. One should also note that the growth temperature of <120 °C is not sufficient to produce copper oxide nanowires. In addition, Cu_2_O with a completely linear structure can be obtained at these reaction temperatures.

**Figure 4:**
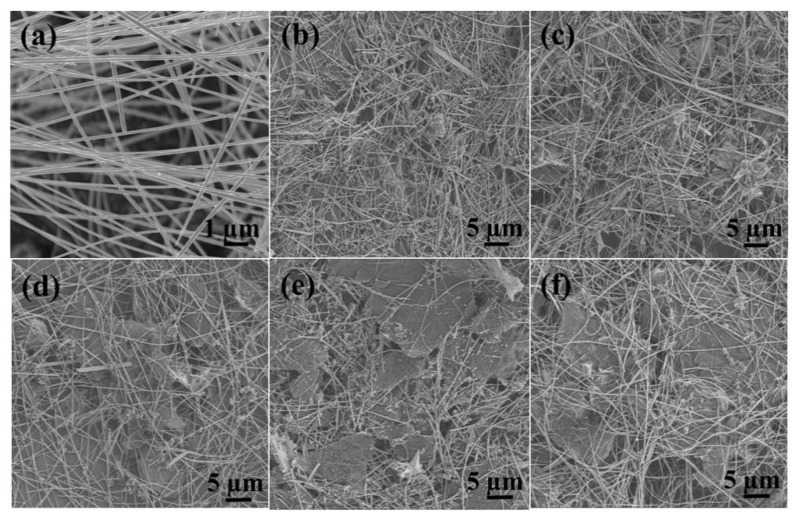
(**a**) SEM image of PPy-coated Cu_2_O nanowires, hydrothermally prepared at 120 °C, and as-prepared rGO/PPy/Cu_2_O nanocomposites after high-temperature thermal reduction. The GO to PPy/Cu_2_O mass ratio is (**b**) 0.08, (**c**) 0.1, (**d**) 0.12, (**e**) 0.15, and (**f**) 0.20.

Furthermore, the graphene content also influences the morphology of resulting nanocomposites. It can be readily observed that graphene facilitates the recombination and coating of PPy/Cu_2_O nanowires. On the other hand, the excessive amount of graphene leads to the stacking of graphene sheets, which is highly undesirable for sensing applications. SEM is utilized to observe the morphology and microstructure of as-prepared rGO/PPy/Cu_2_O nanocomposites (Figure 4). Overall, the utilization ratio of graphene increased with increasing graphene content in as-prepared rGO/PPy/Cu_2_O nanocomposites. However, the excess of graphene will decrease the exposure of PPy-coated Cu_2_O nanowires and result in uneven dispersion and stacking of graphene sheets.

**Figure 5:**
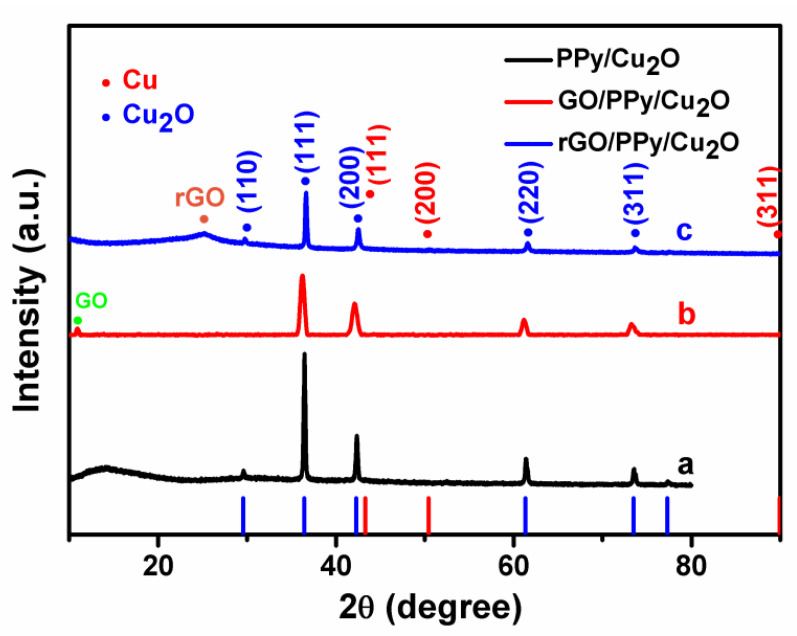
XRD patterns of (a) PPy-coated Cu_2_O, (b) GO/PPy/Cu_2_O nanocomposites and (c) rGO/PPy/Cu_2_O nanocomposites.

The structure of as-prepared nanocomposites was confirmed by X-ray diffraction (XRD). Figure 5 confirms the existence of Cu_2_O and graphene characteristic peaks. Herein, the diffraction peaks at 2θ = 29.5°, 36.4°, 42.2°, 61.3°, and 73.5° correspond to (110), (111), (200), (220), and (311) planes of the Cu_2_O (JCPDS card no. 05-0667) [50,51]. In the XRD patterns of three hybrid structures, we can clearly see the diffraction peaks of Cu_2_O and the (111) and (200) peaks exhibit a relatively high intensity. Similarly, the characteristic diffraction peaks of graphene oxide and reduced graphene oxide are observed at 2θ = 10.2° and 23.1°, respectively. Since the relative quantity of Cu_2_O is much higher than rGO, the diffraction peaks of Cu_2_O are significantly stronger than the rGO. In addition, we have not observed phases other than rGO and Cu_2_O. These results confirm that the rGO/PPy/Cu_2_O nanocomposites have been successfully prepared after high-temperature reduction.

**Figure 6:**
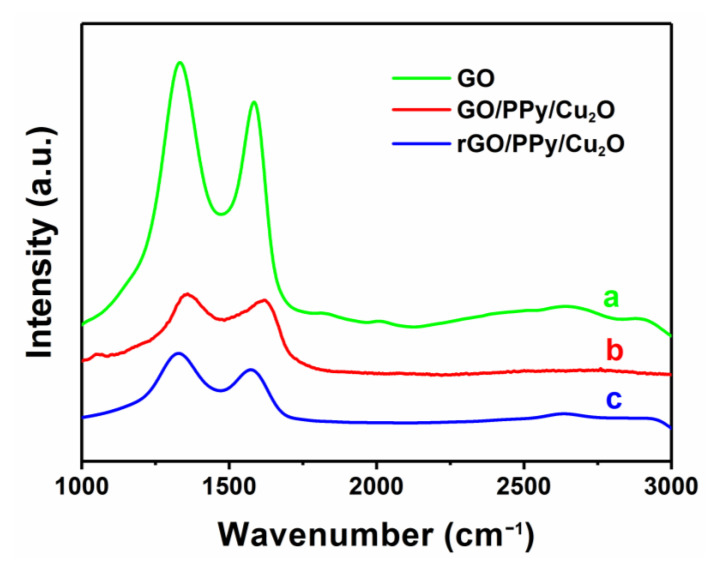
Raman spectra of (a) GO, (b) GO/PPy/Cu_2_O and (c) rGO/PPy/Cu_2_O.

Moreover, Raman spectroscopy is carried out to confirm that the graphene oxide is successfully transformed into the reduced graphene oxide (rGO) [52,53,54,55]. Figure 6 shows two characteristic Raman peaks at 1333 and 1582 cm^−1^, corresponding to D- and G-bands, respectively. The D-band is related to defect scattering and electron/hole recombination during oxidation and reduction processes. Overall, the intensity of D-band represents the degree of disorder in graphene. On the other hand, the G-band is related to the bond stretching of all pairs of sp^2^ atoms, indicating the integrity of sp^2^ hybridized structure. In general, the reduction in graphene is analyzed by measuring the intensity ratio of D- to G-bands (I_D_/I_G_).

**Figure 7:**
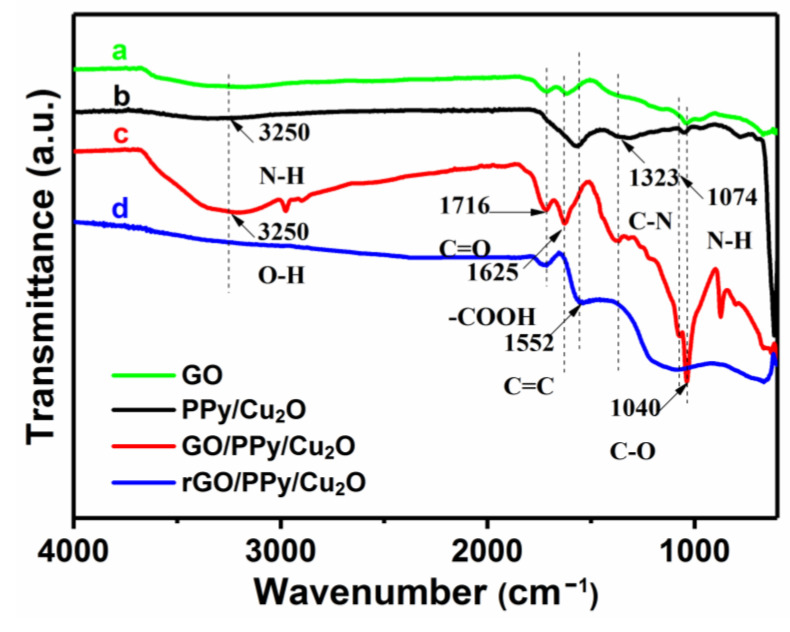
FTIR spectra of (a) GO, (b) PPy-coated Cu_2_O nanowires, (c) GO/PPy/Cu_2_O and (d) rGO/PPy/Cu_2_O nanocomposites.

Figure 6 exhibits that the ID/IG ratio of GO/PPy/Cu_2_O and rGO/PPy/Cu_2_O nanocomposite is 1.133 and 1.153, respectively. Theoretically, when GO is reduced, the oxygen-containing functional groups on the graphene sheets are removed [56], the ordering of sp^2^ carbon network structure is increased, sp^2^ region is widened and the ID/IG ratio is decreased. In fact, a large number of sp^3^ hybridized carbon atoms deoxidize to form a new sp^2^ hybridized region, and the re-formed sp^2^ region is smaller than GO, minimizing the average sp^2^ region of rGO, which is reflected by the enhancement of ID/IG. To further illustrate the successful fabrication of rGO/PPy/Cu_2_O nanocomposites, we have employed Fourier transform infrared spectroscopy (FTIR) to characterize the changes in functional groups before and after high-temperature thermal reduction (Figure 7). The absorption peak near 3250 cm^−1^ can be attributed to N-H stretching vibrations of PPy and O-H stretching vibrations of GO. The absorption peak at 1552 cm^−1^ can be assigned to the vibrations of C=C skeleton, whereas the absorption peaks at 1323 and 1074 cm^−1^ can be attributed to the stretching vibrations of C-N, confirming the existence of PPy in as-prepared rGO/PPy/Cu_2_O nanocomposites. In the case of GO/PPy/Cu_2_O nanocomposite, the absorption peaks at 1625 and 1716 cm^−1^ correspond to the vibrational absorption of -COOH and C=O in carboxylic acids, respectively. One should note that the absorption intensity of -COOH and C=O groups in the FTIR spectrum of rGO/PPy/Cu_2_O nanocomposites is weakened, whereas the absorption peak of C-O at 1040 cm^−1^ is disappeared, indicating the reduction in oxygen-containing functional groups from the graphene surface and confirming the successful transformation of GO into rGO.

**Figure 8:**
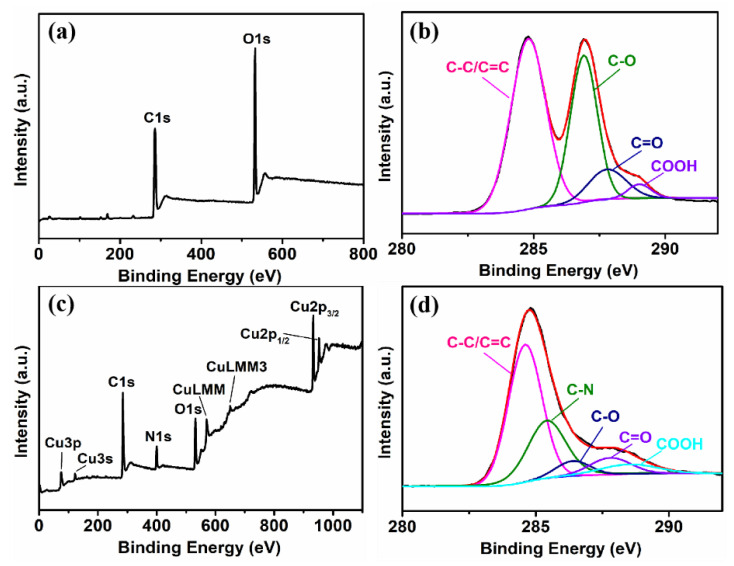
XPS spectra of rGO/PPy/Cu_2_O nanocomposite before and after high-temperature thermal reduction: (**a**) wide-range and (**b**) high-resolution C 1s XPS spectra of GO, and (**c**) wide-range and (**d**) high-resolution C 1s XPS spectra of rGO/PPy/Cu_2_O nanocomposites.

Furthermore, we have utilized X-ray photoelectron spectroscopy (XPS) to qualitatively analyze the elemental composition of as-prepared rGO/PPy/Cu_2_O nanocomposites. Figure 8 shows the wide-range and high-resolution C 1s XPS spectra of GO and rGO/PPy/Cu_2_O. The characteristic peaks of C-C/C=C (284.6 eV), C-O (286.9 eV), C=O (287.8 eV), and COOH (289.0 eV) can be clearly observed in the high-resolution C 1s spectrum (Figure 8b) [57]. The characteristic peak of C-N (285.5 eV) is observed in the high-resolution C 1s spectrum of the as-prepared rGO/PPy/Cu_2_O nanocomposite (Figure 8d). Compared with the graphene oxide, the intensity of C-O, C=O, and COOH peaks is weakened in rGO/PPy/Cu_2_O nanocomposites due to the high-temperature thermal reduction in GO.

Overall, SEM, XRD, Raman spectroscopy. FTIR and XPS confirm the successful synthesis of rGO/PPy/Cu_2_O nanocomposites, confirming the elemental composition and structure.

**Figure 9:**
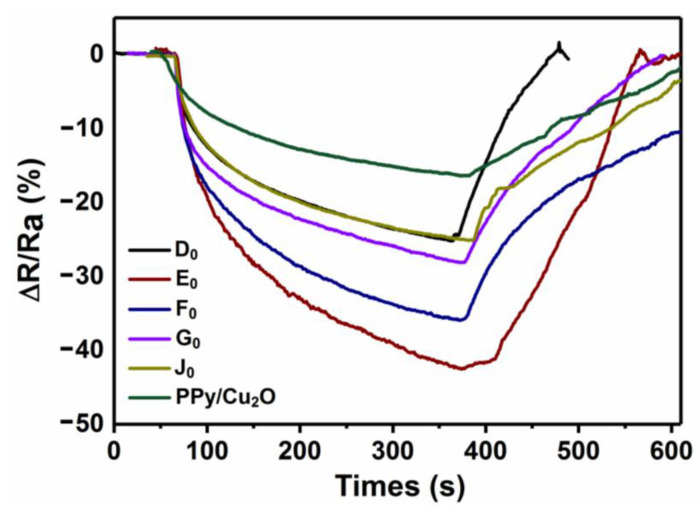
The gas sensing response curves of rGO/PPy/Cu_2_O nanocomposites with different mass ratios of GO to PPy/Cu_2_O nanowires to the NO_2_ flow of 50 ppm (D_0_: 0.08; E_0_: 0.1; F_0_: 0.12; G_0_: 0.15; and J_0_: 0.20).

Figure 9 presents the response curves of PPy-coated Cu_2_O nanowires sensor and rGO/PPy/Cu_2_O nanocomposite sensors, with different graphene contents, to NO_2_ flow of 50 ppm. The mass ratio of GO to PPy/Cu_2_O nanowires was set at 0.08, 0.1, 0.12, 0.15, and 0.2, and the resulting rGO/PPy/Cu_2_O nanocomposites are named as D_0_, E_0_, F_0_, G_0_, and J_0_, respectively. Herein, the resistance response reached the maximum value within 300 sec after the introduction of NO_2_ gas. The gas-sensitive response values of D_0_, E_0_, F_0_, G_0_, and J_0_ were found to be 25.0, 42.5, 35.9, 30.0, and 25.1%, respectively. The ratio of 0.1 composite presents a maximum response, which is about 2.7 times of the sensor based on pure PPy-coated Cu_2_O nanowires (15.7%). Additionally, the sensor recovered the initial resistance level after ≈200 sec under the auxiliary irradiations of an ultraviolet lamp. The experimental results reveal that the rGO/PPy/Cu_2_O-based sensor renders superior gas sensing response, reaching the maximum response value of 42.5% at GO content of E_0_. The further increase in graphene content leads to restacking of graphene sheets and loss of excellent graphene properties, resulting in an inferior gas sensing response.

**Figure 10:**
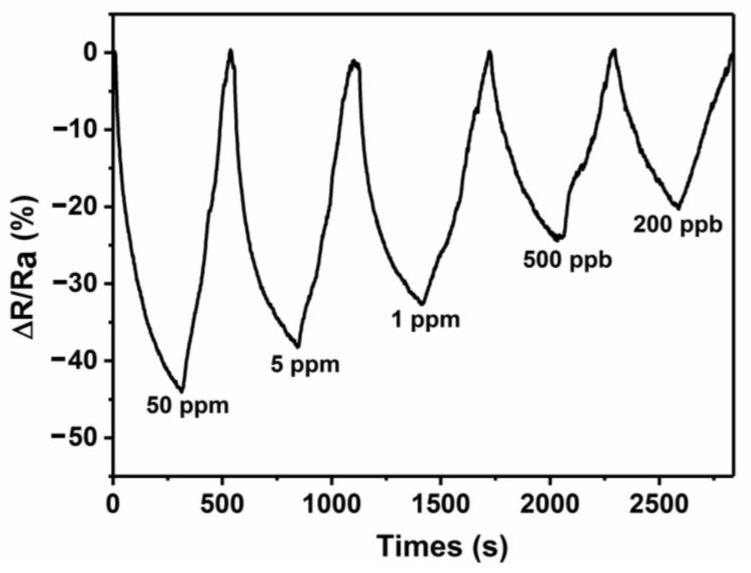
The gas response curves of E_0_-based gas sensor under different NO_2_ concentrations.

As graphene content of 0.1 (E_0_) endows superior gas sensing properties to the as-prepared rGO/PPy/Cu_2_O nanocomposite, we have evaluated the sensing efficiency of E_0_-based gas sensor under different concentrations of NO_2_ (Figure 10). The NO_2_ concentration of 50, 5, 1 ppm, 500 ppb, and 200 ppb resulted in the response value of 44.0, 38.0, 32.7, 24.4, and 20.3%, respectively. Under different gas concentrations, the quick response time of E_0_ is ≈ 300 s and the recovery time can be reduced to ≈150–200 s under auxiliary irradiation of UV lamp [6]. One should note that the E_0_-based gas sensor rendered excellent gas sensing response at low NO_2_ concentrations, which indicates the superior NO_2_ adsorption effect of the as-prepared rGO/PPy/Cu_2_O nanocomposite, resulting in adsorption saturation in a relatively small time and high gas sensitivity. The sensor response with respect to NO_2_ concentration is mainly nonlinear [58,59] because of the Langmuir adsorption of NO_2_ on the surface of active substance. As the concentration of the target gas increases, the adsorption reaches saturation level and results in a decrease in response.

**Figure 11:**
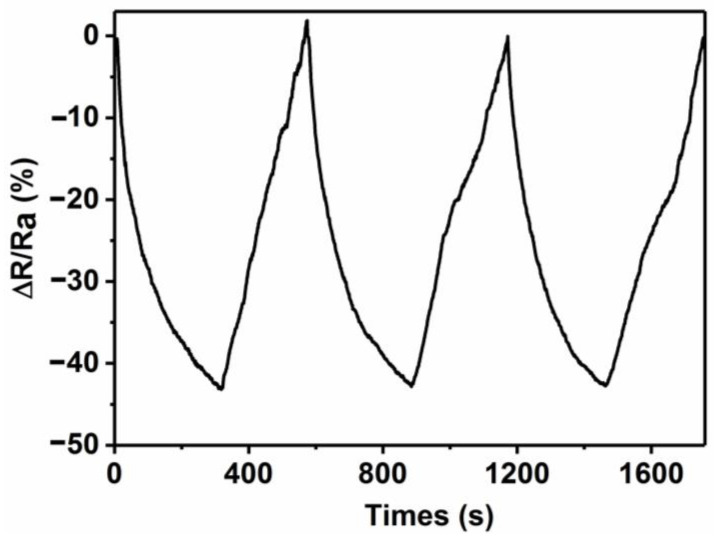
The repeatability and cyclic stability of the E_0_-based gas sensor.

From a practical viewpoint, sustainable reuse is of great significance for gas sensors. Figure 11 shows the repeated gas sensing response evaluation of the E_0_-based gas sensor at NO_2_ flow of 50 ppm, showing excellent repeatability with one cycle consisting of almost 600 s. First of all, the response reaches the saturation level after 300 s of NO_2_ gas injection. Then, the NO_2_ gas is turned-off and background gas is turned-on at the same time. Under the illumination of ultraviolet lamp, NO_2_ gas is gradually desorbed and blown away by air. The sensor begins to gradually recover the initial resistance level. In this way, three cycles of cyclic testing are carried out to detect the repeatability of the E_0_-based gas sensor. Figure 11 confirms that the gas sensing response of the E_0_-based sensor at 50 ppm of NO_2_ gas is stable at ~43.0%. After three cycles, the response sensitivity does not decrease significantly, which further confirms that the as-prepared E_0_-based gas sensor possesses excellent response stability and repeatability. Under normal usage conditions, the GO/PPy/Cu_2_O-based gas sensor demonstrates excellent stability with only a slight decline in the response of 1.5% after 30 days, indicating good long-term stability.

**Figure 12:**
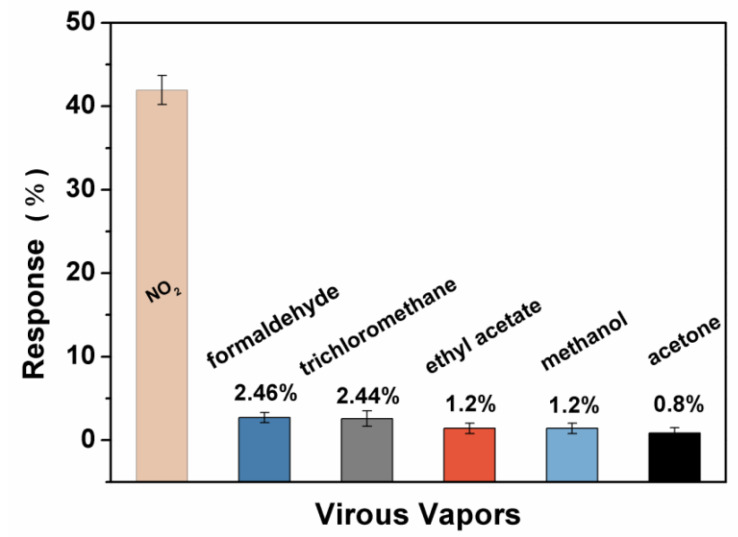
The selectivity of E_0_-based sensor.

Furthermore, it is of utmost importance to assess the selectivity of adsorbed gas in practical applications. Therefore, we have investigated the adsorption of different industrial and laboratory gases, such as chloroform, formaldehyde, ethanol, acetone, and ethyl acetate, by the E_0_-based gas sensor. The saturation vapor pressure of 1% is obtained by the bubbling method and the response of NO_2_ gas (50 ppm) is used as a comparison point to assess sensor selectivity (Figure 12). Figure 12 shows that the response of E_0_-based sensor to other gases is extremely low. For instance, formaldehyde exhibited the highest response of 2.5% among the tested gases, which is much lower than the response of 50 ppm NO_2_ gas (42.5%). One should note that the concentration of these gases is much higher than 50 ppm. Still, the E_0_-based sensor demonstrated superior selectivity to the NO_2_ gas.

### 3.2. Sensing Mechanism

Cu_2_O, rGO, and PPy have similar p-type nature [37,60]. When the composite material is exposed to air, O_2_ molecules could be adsorbed on the material surface in the form of adsorbed

(3) $$O_{2}(\mathrm{gas})\;+{\;e}^{-}\;\to {\;O}_{2}{}^{-}\left(\mathrm{ads}\right)$$

After the introduction of NO_2_, NO_2_ molecules could be directly adsorbed on the surface by capturing electrons from the material (Equation (4)). In addition, NO_2_ also gains electrons from adsorbed oxygen ions (Equation (5)).

(4) $${\mathrm{NO}}_{2}(\mathrm{gas})\;+{\;e}^{-}\;\to {\;\mathrm{NO}}_{2}{}^{-}\left(\mathrm{ads}\right)$$

(5) $${\mathrm{NO}}_{2}(\mathrm{gas})\;+{\;O}_{2}{}^{-}(\mathrm{ads})\;+{\;2e}^{-}\;\to {\;\mathrm{NO}}_{2}{}^{-}(\mathrm{ads})\;+{\;2O}^{-}$$

**Figure 13:**
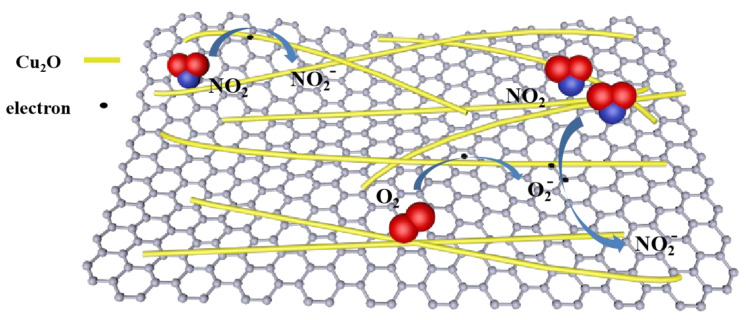
Schematic illustration of the NO_2_ sensing mechanism of rGO/PPy/Cu_2_O-based sensor.

Figure 13 illustrates the gas sensing mechanism. After the above process [61], the hole concentration of the device increases. Herein, p-type polypyrrole completes the process of doping and de-doping by gas adsorption and desorption, respectively [62,63]. Meanwhile, graphene and polypyrrole provide a large number of binding sites for gas adsorption. The high charge mobility of conducting polymer, i.e., polypyrrole, and graphene facilitates carrier transport and migration to the electrode for collection. These processes lead to the rapid decrease in electron concentration within the rGO/PPy/Cu_2_O composites. In general, hole-assisted carrier transport is responsible for the conduction of p-type semiconductors. The hole concentration significantly increases with the decrease in electron concentration in rGO/PPy/Cu_2_O composite due to NO_2_ adsorption, which increases sensor conductivity.

According to the principle of complementary feedback of gas sensor [64], the combination of p-type semiconductors in gas sensors renders a synergistic influence on gas sensing characteristics and temperature coefficients of both materials, reducing zero drift, shortening initial relaxation time, and rendering superior selectivity and stability. Herein, the interdigital electrode is equivalent to the parallel connection of a sensor and several resistors, which reduces the initial resistance of the sensor. The decrease in initial resistance of sensor increased the change in resistance, which corresponds to the response value. During the recovery stage of gas sensor, the newly adsorbed air molecules eliminate residual NO_2_ molecules from the surface of rGO/PPy/Cu_2_O nanocomposite by introducing air and auxiliary irradiations under an ultraviolet lamp [65], increasing the resistivity of p-type semiconductor and recovering to the initial resistance.

## 4. Conclusions

In summary, PPy-coated Cu_2_O nanowires have been prepared by the hydrothermal reaction and combined with graphene oxide to obtain rGO/PPy/Cu_2_O nanocomposites after high-temperature thermal reduction. Moreover, a p-p-type gas sensor has been fabricated using rGO/PPy/Cu_2_O nanocomposite as an electrode and room temperature sensing is realized. The results revealed that the rGO/PPy/Cu_2_O-based gas sensor renders better NO_2_ sensing performance than the PPy/Cu_2_O-based sensor, confirming the positive influence of graphene addition. When the mass ratio of graphene to PPy-coated Cu_2_O nanowires was 0.1, the rGO/PPy/Cu_2_O-based sensor demonstrated the highest response value of 42.5% for NO_2_ gas (50 ppm). When the concentration of NO_2_ was as low as 200 ppb, the rGO/PPy/Cu_2_O-based sensor still exhibited a response value of 20.3%. Moreover, the rGO/PPy/Cu_2_O-based sensor has also rendered stable repeatability and excellent selectivity at the NO_2_ concentration of 50 ppm.

## Figures and Tables

**Table 1:** The comparison of various NO_2_ sensors based on rGO and Cu_2_O composites.

Material	Response	Concentration	Working Temperature	Reference
SnO_2_/graphene	0.25 (∆R/R_a_)	10 ppm	Room temperature	[6]
rGO-SnO_2_	53.57 (R_g_/R_a_)	3 ppm	125 °C	[22]
SnS_2_-rGO	9.8% (∆R/R_a_)	0.6 ppm	80 °C	[27]
rGO/In_2_O_3_	22.3 (R_g_/R_a_)	500 ppb	150 °C	[28]
BiVO_4_/Cu_2_O	4.2 (R_g_/R_a_)	4 ppm	60 °C	[29]
BiVO_4_/Cu_2_O/rGO	8.2 (R_g_/R_a_)	1 ppm	60 °C	[30]
Au/Cu_2_O/ZnO	26% (∆R/R_a_)	5 ppb	Room temperature	[31]
MoS_2_/graphene	69% (∆R/R_a_)	10 ppm	200 °C	[33]

## Data Availability

Not applicable.
